# Correction to: 33rd Annual Meeting & Pre-Conference Programs of the Society for Immunotherapy of Cancer (SITC 2018)

**DOI:** 10.1186/s40425-019-0519-y

**Published:** 2019-02-13

**Authors:** Sneha Berry, Nicolas Giraldo, Peter Nguyen, Benjamin Green, Haiying Xu, Aleksandra Ogurtsova, Abha Soni, Farah Succaria, Daphne Wang, Charles Roberts, Julie Stein, Elizabeth Engle, Drew Pardoll, Robert Anders, Tricia Cottrell, Janis M. Taube, Ben Tran, Mark Voskoboynik, James Kuo, Yung-Lue Bang, Hyun-Cheo Chung, Myung-Ju Ahn, Sang-We Kim, Ayesh Perera, Daniel Freeman, Ikbel Achour, Raffaella Faggioni, Feng Xiao, Charles Ferte, Charlotte Lemech, Funda Meric-Bernstam, Theresa Werner, Stephen Hodi, Wells Messersmith, Nancy Lewis, Craig Talluto, Mirek Dostalek, Aiyang Tao, Sarah McWhirter, Damian Trujillo, Jason Luke, Chunxiao Xu, BoMarelli, Jin Qi, Guozhong Qin, Huakui Yu, Molly Jenkins, Kin-Ming Lo, Joern-Peter Halle, Yan Lan, Matthew Taylor, Nicholas Vogelzang, Allen Cohn, Daniel Stepan, Robert Shumaker, Corina Dutcus, Matthew Guo, Emmett Schmidt, Drew Rasco, Marcia Brose, Nicholas Vogelzang, Christopher Di Simone, Sharad Jain, Donald Richards, Carlos Encarnacion, Drew Rasco, Robert Shumaker, Corina Dutcus, Daniel Stepan, Matthew Guo, Emmett Schmidt, Matthew Taylor, Nicholas Vogelzang, Carlos Encarnacion, Allen Cohn, Christopher Di Simone, Drew Rasco, Donald Richards, Matthew Taylor, Corina Dutcus, Daniel Stepan, Robert Shumaker, Matthew Guo, Emmett Schmidt, James Mier, Jeongshin An, Yeun-yeoul Yang, Won-Hee Lee, Jinho Yang, Jong-kyu Kim, Hyun Goo Kim, Se Hyun Paek, Jun Woo Lee, Joohyun Woo, Jong Bin Kim, Hyungju Kwon, Woosung Lim, Nam Sun Paik, Yoon-Keun Kim, Byung-In Moon, Filip Janku, David Tan, Juan Martin-Liberal, Shunji Takahashi, Ravit Geva, Ayca Gucalp, Xueying Chen, Kulandayan Subramanian, Jennifer Mataraza, Jennifer Wheler, Philippe Bedard

**Affiliations:** 1 Johns Hopkins University School of Medicine, Baltimore, MD USA; 2 MedImmune, Gaithersburg, MD USA; 3 Peter MacCallum Cancer Center, Melbourne, Australia; 4 Nucleus Network, Melbourne, Australia; 5 Scientia Clinical Research, Sydney, Australia; 6 Seoul National University Hospital, Seoul, Korea, Republic of; 7 Yonsei Cancer Center, Yonsei University, Seoul, Korea, Republic of; 8 Samsung Medical Center, Seoul, Korea, Republic of; 9 Asan Medical Center, Songpa-Gu, Korea, Republic of; 10 MD Anderson Cancer Center, Houston, TX USA; 11 University of Utah, Salt Lake City, UT USA; 12 Dana-Faber Cancer Institute, Boston, MA USA; 13 University of Colorado Cancer Center, Aurora, CO USA; 14 Novartis Pharmaceuticals Corporation, East Hanover, NJ USA; 15 Novartis Institutes for BioMedical Resea, Cambridge, MA USA; 16 Aduro Biotech Inc, Berkeley, CA USA; 17 The University of Chicago Medicine, Chicago, IL USA; 18 EMD Serono Research and Development, Belmont, MA USA; 19 Oregon Health and Science University, Portland, OR USA; 20 McKesson Specialty Health, Las Vegas, NV USA; 21 Eisai Inc., Woodcliff Lake, NJ USA; 22 Merck & Co., Inc., Kenilworth, NJ USA; 23 South Texas Accelerated Research Therape, San Antonio, TX USA; 24 Abramson Cancer Center of the University, Philadelphia, PA USA; 25 McKesson Specialty Health, Las Vegas, NV USA; 26 South Texas Accelerated Research Therape, San Antonio, TX USA; 27 Eisai Inc., Woodcliff Lake, NJ USA; 28 Merck & Co., Inc., Kenilworth, NJ USA; 29 Oregon Health and Science University, Portland, OR USA; 30 McKesson Specialty Health, Las Vegas, NV USA; 31 South Texas Accelerated Research Therape, San Antonio, TX USA; 32 Oregon Health and Science University, Portland, OR USA; 33 Eisai Inc., Woodcliff Lake, NJ USA; 34 Merck & Co., Inc., Kenilworth, NJ USA; 35 Beth Israel Deaconess Medical Center, Boston, MA USA; 36 Ewha Womans University, Seoul, Korea, Republic of; 37 MD healthcare company, Seoul, Korea, Republic of; 38 MD Anderson Cancer Center, Houston, TX USA; 39 National University Cancer Institute, Singapore, Singapore; 40 Vall d’Hebron Institute of Oncology, Barcelona, Spain; 41 The Cancer Institute Hospital of JFCR, Tokyo, Japan; 42 Tel Aviv Sourasky Medical Center, Tel-Aviv, Israel; 43 Memorial Sloan Kettering Cancer Center, New York, NY USA; 44 Novartis Pharmaceuticals Corporation, East Hanover, NJ USA; 45 Novartis Institutes for BioMedical Resea, Cambridge, MA USA; 46 Princess Margaret Cancer Centre, Toronto, ON Canada

**Correction to: J Immuno Therapy Cancer. (2018) 6 (Suppl 1)**

**https://doi.org/10.1186/s40425-018-0422-y**

**https://doi.org/10.1186/s40425-018-0423-x**

After publication of this supplement [1, 2], it was brought to our attention that due to an error authors were either missing or incorrectly indicated as contributing author while in fact they didn't contribute in the following abstracts. In addition affiliations were listed where in fact there were no contributing authors affiliated with them in the following abstracts. This has now been included in this correction.

**P128**

**Multiplexed immunofluorescent assay development for study of the PD-1/PD-L1 checkpoint in the tumor immune microenvironment (TIME)**

Sneha Berry, MS, Nicolas Giraldo, MD PhD, Peter Nguyen, MS, Benjamin Green, BS, Haiying Xu, Aleksandra Ogurtsova, Abha Soni, DO, Farah Succaria, MD, Daphne Wang, MS, Charles Roberts, Julie Stein, MD, Elizabeth Engle, MSc, Drew Pardoll, MD, PhD, Robert Anders, MD, PhD, Tricia Cottrell, MD, PhD, Janis M. Taube, MD

Johns Hopkins University School of Medicine, Baltimore, MD, USA

**Correspondence:** Sneha Berry (jtaube1@jhmi.edu)

*Journal for ImmunoTherapy of Cancer* 2018, **6(Suppl 1)**:P128

**Janis M. Taube, MD is a contributing author and has therefore been added to the author list in this correction article. Contributing author Sneha Berry, MS is listed three times in the original article; this is no longer the case in this correction article.**

**Background**

Multispectral immunofluorescent (mIF) staining of formalin-fixed paraffin-embedded (FFPE) tissue allows spatially-resolved quantitative analysis of cell position and protein expression. The design and validation of mIF panels is a challenge. Our goal was to develop a 7-plex assay for characterizing PD-1 and PD-L1 expression, with high sensitivity for multiple markers and minimal bleed-through between fluorescent channels, while avoiding steric hindrance among markers occupying the same cellular compartment.

**Methods**

Single IF slides were stained for PD-1, PD-L1, CD8, FoxP3, CD163, and a tumor marker (e.g. Sox10/S100 for melanoma) using primary antibodies at manufacturer’s recommended concentrations and visualized with an Opal kit (PerkinElmer). Positive signal was compared to chromogenic IHC (n=3 tonsil specimens). In some instances, the kit’s HRP-polymer was substituted for one that provided greater amplification. Primary antibody titrations were performed, and the concentration with comparable signal to chromogenic IHC that showed the highest IF signal to noise ratio was selected. Using the selected primary antibody concentration, TSA dilution series were performed on n=5 tumor specimens to minimize bleed-through. Finally, the optimized single IF stains were combined into multiplex format, which was again validated to ensure no positivity loss. Images were scanned with the Vectra 3.0 and processed using inForm (Ver 2.3).

**Results**

The percent positive pixels for CD163, CD8, and tumor marker expression by IF were comparable to chromogenic IHC with manufacturer’s recommended protocols (p>0.05). However, PD-1, PD-L1, and FoxP3 showed ~50% loss of signal (p<0.05), which was recovered by replacing the Opal kit's secondary HRP polymer with PowerVision (Leica). Unbalanced fluorescence intensities between 540 to 570 Opal dyes resulted in significant bleed-through and led to false positive pixels. This error was minimized >2 fold (2.5% to 1.1%) by concentrating the 570 dye and ensuring that this dye pair was used to study markers in different cellular compartments (nuclear FoxP3 vs. membrane CD8), so any residual bleed-through could be discounted during image analysis. Using the optimized panel, we are able to reliably identify cell types contributing PD-L1 and PD-1 to the TIME, and even resolve populations of PD-1^high^ vs. PD-1^low^ lymphocytes.

**Conclusions**

We demonstrate successful optimization of a 7-color multiplex panel characterizing the PD-1/PD-L1 axis to provide high quality data sets for whole slide or regional analysis of the TIME. With the use of multiparametric assays such as this, we hope to guide improved approaches to patient selection and potentially identify additional tumor types likely to respond to anti-PD-(L)1 immunotherapy.

**Ethics Approval**

The study was approved by Johns Hopkins University Institutional Review Board.

**P308**

**A Phase 1 study of MEDI5752, a bispecific antibody that preferentially targets PD-1 and CTLA-4 expressing T cells, in patients with advanced solid tumors**

Ben Tran^2^, Mark Voskoboynik^3^, James Kuo^4^, Yung-Lue Bang^5^, Hyun-Cheo Chung^6^, Myung-Ju Ahn^7^, Sang-We Kim^8^, Ayesh Perera^1^, Daniel Freeman^1^, Ikbel Achour^1^, Raffaella Faggioni^1^, Feng Xiao^1^, Charles Ferte^1^, Charlotte Lemech^4^

^1^MedImmune, Gaithersburg, MD, USA; ^2^Peter MacCallum Cancer Center, Melbourne, Australia; ^3^Nucleus Network, Melbourne, Australia; ^4^Scientia Clinical Research, Sydney, Australia; ^5^Seoul National University Hospital, Seoul, Korea, Republic of; ^6^Yonsei Cancer Center, Yonsei University, Seoul, Korea, Republic of; ^7^Samsung Medical Center, Seoul, Korea, Republic of; ^8^Asan Medical Center, Songpa-Gu, Korea, Republic of

**Correspondence**: Charles Ferte (fertec@MedImmune.com*) Journal for ImmunoTherapy of Cancer* 2018, **6(Suppl 1)**:P308

**Jeff Brubaker was not a contributing author and has therefore been removed from the author list in this correction article. The credentials as shown on the original article are no longer listed on this correction article.**

**Background**

Based on demonstrated clinical activity and manageable safety profiles, checkpoint inhibiting antibodies blocking PD 1, PD-L1, or CTLA-4 have received regulatory approvals for the treatment of various malignancies [1-5]. The combination therapy with anti-PD-1 and anti-CTLA-4 agents is approved by FDA for metastatic melanoma, renal cell carcinoma and microsatellite instability-high (MSI-H) or mismatch repair deficient (dMMR) metastatic colorectal cancer, based on improved overall survival versus either agent alone [6-10]. Numerous clinical studies of combination immunotherapy are currently investigating the same combination across a range of solid tumors [11- 15]. Although the efficacy of these drug combinations is dose dependent, the toxicity associated with anti-CTLA-4 agents, in particular, is dose limiting, thereby potentially affecting treatment outcomes with combination therapy.- MEDI5752 is a bispecific humanized IgG1 monoclonal antibody that binds PD-1 and CTLA-4. In contrast to the combination therapy, MEDI5752 exhibits a novel T cell targeting mechanism that could provide a favorable toxicity profile. In addition, we have shown that MEDI5752 can impact cell surface expression of PD-1. Based on these novel mechanisms of action, MEDI5752 may show improved efficacy and safety in comparison to co- administration of conventional anti-PD1/anti-PD-L1 and anti-CTLA-4 antibodies.

**Methods**

This is a Phase 1, first-time-in-human, multicenter, open-label study in patients with advanced solid tumors. The dose-escalation phase will evaluate approximately six MEDI5752 dose levels to identify a maximum tolerated dose. Dose escalation will be followed by two dose-expansion cohorts in defined setting with patients with advanced or metastatic solid tumor and tested against a control arm. Subjects will remain on treatment until confirmed progressive disease, initiation of alternative cancer therapy, unacceptable toxicity, or other reason for discontinuation. The primary endpoints are safety and efficacy (objective response in the dose-expansion phase). Secondary endpoints include additional efficacy assessment across both phases, pharmacokinetics, and immunogenicity.

**Trial Registration**

NCT03530397

**References**

1. Opdivo Prescribing Information. Princeton, NJ: Bristol-Myers Squibb Company, 2018. [revised 2018 Apr].

2. Keytruda Prescribing Information. Whitehouse Station, NJ: Merck Sharp & Dohme Corp, 2018 [revised 2018 Jun].

3. Tecentriq Prescribing Information. San Francisco (CA): Genentech, Inc, 2018. [revised 2019 Jul].

4. Imfinzi Prescribing Information. Wilmington (DE): AstraZeneca Pharmaceuticals LP, 2018. [revised 2018 Feb].

5. Yervoy Prescribing Information. Princeton (NJ): Bristol-Myers Squibb Company, 2018. [revised 2018 Apr].

6. Larkin J, Chiarion-Sileni V, Gonzalez R, Grob JJ, Cowey CL, Lao CD, et al. Combined nivolumab and ipilimumab or monotherapy in untreated melanoma. N Engl J Med. 2015;373:23-34.

7. Postow MA, Chesney J, Pavlick AC, Robert C, Grossmann K, McDermott D, et al. Nivolumab and ipilimumab versus ipilimumab in untreated melanoma. N Engl J Med. 2015;372:2006-2017.

8. Motzer RJ, Tannir NM, McDermott DF, Frontera OA, Melichar B, Choueiri TK, et al. Nivolumab plus ipilimumab versus sunitinib in advanced renalcell carcinoma. N Engl J Med. 2018;378:1277-1290.

9. Overman MJ, Lonardi S, Wong KYM, Lenz H-J, Gelsomino F, Aglietta M, et al. Durable clinical benefit with nivolumab plus ipilimumab in DNA mismatch repair–deficient/microsatellite instability–high metastatic colorectal cancer. J Clin Oncol. 2018;36:773-779.

10. Broderick JM. Nivolumab, ipilimumab combo approved by FDA for MSI-H/dMMR colorectal cancer. Pharmacy Times, 11 July, 2018. Available at: https://www.pharmacytimes.com/news/nivolumab-ipilimumabcombo-approved-by-fda-for-msihdmmr-colorectal-cancer. Accessed 18 July, 2018.

11. Antonia S, Goldberg SB, Balmanoukian A, Chaft JE, Sanborn RE, Gupta A, et al. Safety and antitumour activity of durvalumab plus tremelimumab in non-small cell lung cancer: a multicentre, phase 1b study. Lancet Oncol. 2016a;17(3):299-308.

12. Haddad R, Gillison M, Ferris RL, Harrington K, Monga M, Baudelet C, et al. Double-blind, two-arm, phase 2 study of nivolumab (nivo) in combination with ipilimumab (ipi) versus nivo and ipi-placebo (PBO) as first-line (1L) therapy in patients (patients) with recurrent or metastatic squamous cell carcinoma of the head and neck (R/M SCCHN)—CheckMate 714. Ann Oncol. 2016;27(suppl_6):1017TiP-TiP.

13. Kelley RK, Abou-Alfa GK, Bendell JC, Kim T-Y, Borad MJ, Yong W-P, et al. Phase I/II study of durvalumab and tremelimumab in patients with unresectable hepatocellular carcinoma (HCC): Phase I safety and efficacy analyses. J Clin Oncol. 2017;35(15_suppl):4073.

14. Janjigian YY, Ott PA, Calvo E, Kim JW, Ascierto PA, Sharma P, et al. Nivolumab ± ipilimumab in patients with advanced (adv)/metastatic chemotherapy-refractory (CTx-R) gastric (G), esophageal (E), or gastroesophageal junction (GEJ) cancer: CheckMate 032 study. J Clin Oncol. 2017;35(15_suppl):4014.

15. Escudier B, Tannir NM, McDermott DF, Frontera OA, Melichar B, Plimack ER, et al. LBA5CheckMate 214: Efficacy and safety of nivolumab + ipilimumab (N+I) v sunitinib (S) for treatment-naïve advanced or metastatic renal cell carcinoma (mRCC), including IMDC risk and PD-L1 expression subgroups. Ann Oncol. 2017;28(suppl_5):mdx440.029- mdx440.029.

**P309**

**Phase I dose-finding study of MIW815 (ADU-S100), an intratumoral STING agonist, in patients with advanced solid tumors or lymphomas**

Funda Meric-Bernstam, MD^2^, Theresa Werner^3^, Stephen Hodi^4^, Wells Messersmith, MD^5^, Nancy Lewis^6^, Craig Talluto^7^, Mirek Dostalek^6^, Aiyang Tao^6^, Sarah McWhirter^8^, Damian Trujillo^8^, Jason Luke, MD, FACP^9^

^2^MD Anderson Cancer Center, Houston, TX, USA; ^3^University of Utah, Salt Lake City, UT, USA; ^4^Dana-Faber Cancer Institute, Boston, MA, USA; ^5^University of Colorado Cancer Center, Aurora, CO, USA; ^6^Novartis Pharmaceuticals Corporation, East Hanover, NJ, USA; ^7^Novartis Institutes for BioMedical Resea, Cambridge, MA, USA; ^8^Aduro Biotech Inc, Berkeley, CA, USA; ^9^The University of Chicago Medicine, Chicago, IL, USA

**Correspondence:** Funda Meric-Bernstam (fmeric@mdanderson.org)

*Journal for ImmunoTherapy of Cancer* 2018, **6(Suppl 1)**:P309

**Janis Callister was not a contributing author and has therefore been removed from the author list in this correction article. The redundant affiliation Articulate Science as shown on the original article is no longer listed on this correction article.**

**Background**

MIW815 (ADU-S100) is a novel synthetic cyclic dinucleotide that can activate human STING (STimulator of INterferon Genes) in antigenpresenting cells. In preclinical models, STING pathway activation can induce tumor antigen-specific T-cell priming within the tumor microenvironment, leading to antitumor immunity and tumor destruction.

**Methods**

Eligible patients (≥2 accessible tumors; Eastern Cooperative Oncology Group Performance Status ≤1) include those with advanced/metastatic solid tumors or lymphomas with progressive disease despite standard of care or for whom there is no standard treatment.

MIW815 (ADU-S100) is administered by weekly intratumoral injections (3 weeks on/1 week off) at escalating doses (starting dose: 50μg) in 28-day cycles. Primary objectives are to characterize safety and tolerability and to identify a recommended dose for future studies.

Secondary objectives include preliminary efficacy, pharmacokinetics (PK), and pharmacodynamics (PD). The study is currently in dose escalation.

**Results**

As of June 15, 2018, 41 heavily pretreated patients (median age 62 years; range 26–80 years) with various solid tumors or lymphomas were enrolled. Thirty-five patients have discontinued from the study for the following reasons: disease progression (n=26), physician/patient decision (n=8), and death (n=1); 6 patients continue to receive treatment. No dose-limiting toxicities (DLTs) were reported during the first cycle at any dose level. The most common (≥10% of patients) treatment-related AEs (TRAEs) were pyrexia (n=7; 17.1%), injection site pain (n=6; 14.6%), and headache (n=6; 14.6%). Grade 3/4 TRAEs included increased lipase (n=2; 4.9%) and elevated amylase, tumor pain, dyspnea, respiratory failure, and injection site reaction (n=1 each; 2.4%). Systemic MIW815 (ADU- S100) exposure increased with dose. On-treatment tumor biopsies showed increases in CD8 T cells infiltrating the injected tumors in a subset of patients. Preliminary antitumor activity, PK analysis, and PD data from injected lesions, noninjection lesions, and peripheral blood, will be presented.

**Conclusions**

Intratumoral injection of MIW815 (ADU-S100) was well tolerated in doses tested thus far in patients with advanced solid tumors and lymphoma, with no DLTs reported to date. Trials evaluating combinations of MIW815 (ADU-S100) with anti-PD1 or anti-CTLA4 antibodies are ongoing.

**Ethics Approval**

This study was approved by an independent ethics committee or institutional review board at each site.

**P383**

**Combination therapy with M7824 (MSB0011359C) and NHSmuIL12 enhances antitumor efficacy in preclinical cancer models**

Chunxiao Xu, PhD^2^, Bo Marelli^2^, Jin Qi^2^, Guozhong Qin^2^, Huakui Yu^2^, Molly Jenkins^2^, Kin-Ming Lo^2^, Joern-Peter Halle^2^, Yan Lan, MD^2^

^2^EMD Serono Research and Development, Belmont,

MA, USA

**Correspondence:** Yan Lan (yan.lan@emdserono.com)

*Journal for ImmunoTherapy of Cancer* 2018, **6(Suppl 1)**:P383

**Colleen Stanton was not a contributing author and has therefore been removed from the author list in this correction article. The redundant affiliation Nucleus Global as shown on the original article is no longer listed on this correction article.**

**Background**

PD-1/PD-L1 pathway inhibition is a clinically validated approach in cancer therapy. However, most patients do not respond to the monotherapy due to multiple immunosuppressive mechanisms. Combining anti-PD-1/ PD-L1 with other immunotherapeutic agents targeting additional immunomodulatory pathways in the tumor microenvironment (TME) is one strategy to overcome resistance and improve response rates. M7824 is an innovative first-in-class bifunctional fusion protein composed of two extracellular domains of TGF-β receptor II (a TGF-β “trap”) fused to a human anti-PD-L1 IgG1 monoclonal antibody. Through simultaneous blockade of the PD-L1 and TGF-β pathways, M7824 demonstrated enhanced anti-tumor activity in preclinical models [1]. NHS-IL12, and the surrogate NHS- muIL12, are immunocytokines designed to target tumor necrotic regions to deliver IL-12 into the TME, where they can activate NK cells and CD8+ T cells to increase their cytotoxic functions. The surrogate NHSmuIL12 has demonstrated antitumor efficacy in preclinical models [2].

This study is designed to investigate whether M7824 treatment may further benefit from combination therapy with NHS-muIL12.

**Methods**

Mice bearing MC38, EMT-6, or 4T1 tumors were treated with M7824, NHS-muIL12, or combination therapy. Tumor growth and survival were assessed in each model, and tumor recurrence following remission and rechallenge was evaluated in the EMT-6 model. Immune cell populations in the spleens and tumors were evaluated by flow cytometry and the frequency of tumor antigen-reactive IFNγ-producing CD8+ T cells was evaluated by an ELISpot assay in the MC38 model.

**Results**

Combination of M7824 and NHS-muIL12 enhanced antitumor activity and extended the survival relative to either monotherapy in preclinical tumor models. Combination therapy also enhanced the proliferation, infiltration, and cytotoxicity of CD8+ T cells relative to monotherapies. In addition, the combination therapy increased the frequency of tumor antigenreactive T cells and induced the generation of tumor-specific immune memory, as demonstrated by protection against tumor rechallenge.

**Conclusions**

These data demonstrate that combination therapy with M7824 and NHS-muIL12 improved anti-tumor efficacy in multiple preclinical tumor models and suggest that combining these therapies may be a promising therapeutic strategy for patients with solid tumors.

**References**

1. Lan Y, et al. Enhanced preclinical antitumor activity of M7824, a bifunctional fusion protein simultaneously targeting PD-L1 and TGF-β. Sci Trans Med. 2018;10(424).

2. Fallon J, et al. The immunocytokine NHS-IL12 as a potential cancer therapeutic. Oncotarget 2014;5(7):1869-84.

**P391**

**A phase 1b/2 trial of lenvatinib in combination with pembrolizumab in patients with advanced melanoma**

Matthew Taylor, MD^2^, Nicholas Vogelzang, MD^3^, Allen Cohn^3^, Daniel Stepan^4^, Robert Shumaker, PhD,^4^, Corina Dutcus^4^, Matthew Guo^4^, Emmett Schmidt, MD PhD^5^, Drew Rasco^6^

^2^Oregon Health and Science University, Portland, OR, USA; ^3^McKesson Specialty Health, Las Vegas, NV, USA; ^4^Eisai Inc., Woodcliff Lake, NJ, USA; ^5^Merck & Co., Inc., Kenilworth, NJ, USA; ^6^South Texas Accelerated Research Therape, San Antonio, TX, USA

**Correspondence:** Matthew Taylor (taylmatt@ohsu.edu) *Journal for ImmunoTherapy of Cancer* 2018, **6(Suppl 1)**:P391

**The redundant affiliation 1. Oxford PharmaGenesis, Oxford, UK as shown on the original article is no longer listed on this correction article.**

**Background**

Lenvatinib is a multikinase inhibitor of VEGFR 1−3, FGFR 1−4, PDGFRα, RET, and KIT. Pembrolizumab, an anti-PD-1 antibody, is approved for the first-line treatment of patients with advanced melanoma, with objective response rates (ORR) of 21–34% [1,2]. Preclinical studies indicate that lenvatinib decreases the population of tumorassociated macrophages, increases CD8+ T cell infiltration, and augments the activity of PD-1 inhibitors; therefore, lenvatinib is a rational combination partner for pembrolizumab [3,4]. We report interim results of an ongoing phase 1b/2 trial evaluating lenvatinib in combination with pembrolizumab in patients with solid tumors, focusing on the advanced melanoma cohort.

**Methods**

In this multicenter, open-label study (NCT02501096), patients with measurable, confirmed, metastatic melanoma and ECOG performance status ≤1 received lenvatinib (20 mg/day orally) + pembrolizumab (200 mg Q3W, IV). Patients were not preselected based on PDL1 status. Tumor assessments were performed by study investigators using immune-related RECIST (irRECIST). The phase 2 primary end point was ORR at 24 weeks (ORRWK24). Secondary end points included ORR, progression-free survival (PFS), and duration of response (DOR).

**Results**

At the data cutoff of March 1, 2018, 21 patients were enrolled: 14 (67%) patients were PD-L1(+), 4 (19%) were PD-L1(-), 3 (14%) were not tested; and 38% of patients had ≥1 prior anticancer therapy. The primary end point of ORRWK24 was 47.6% (95% CI, 25.7–70.2). Additional efficacy outcomes are summarized in the table (Table 1). All patients experienced ≥1 treatment-related adverse event (TRAE). Grade 3 and 4 TRAEs occurred in 13 (62%) and 1 (5%; adrenal insufficiency) patients respectively. There were no fatal TRAEs. The most common any-grade TRAEs were fatigue (52%), decreased appetite (48%), diarrhea (48%), hypertension (48%), dysphonia (43%), and nausea (43%). Dose reduction and interruption due to TRAEs occurred in 13 (62%) and 10 (48%) patients, respectively.

**Conclusions**

The lenvatinib and pembrolizumab combination regimen was welltolerated and demonstrated encouraging clinical activity. The combination may potentially improve upon the antitumor activity of antiPD-1 monotherapies, supporting further evaluation of this regimen in patients with advanced melanoma.

**Trial Registration**

NCT02501096

**References**

1. Ribas A, et al. Pembrolizumab versus investigator-choice chemotherapy for ipilimumab-refractory melanoma (KEYNOTE-002): a randomised, controlled, phase 2 trial. Lancet Oncology. 2015;16(8):908-18.

2. Robert C, et al. Pembrolizumab versus ipilimumab in advanced melanoma. N Engl J Med. 2015;372(26):2521- 2532.

3. Kato Y et al. Effects of lenvatinib on tumor-associated macrophages enhance antitumor activity of PD-1 signal inhibitors. Mol Cancer Ther. 2015;14 (12Suppl2):A92.

4. Kato Y. Upregulation of memory T cell population and enhancement of Th1 response by lenvatinib potentiate antitumor activity of PD-1 signaling blockade. Cancer Res. 2017;77 (13 Suppl):4614. Ethics Approval This study was approved by all relevant institutional review boards.

**Table 1 (abstract P391)**. See text for description.

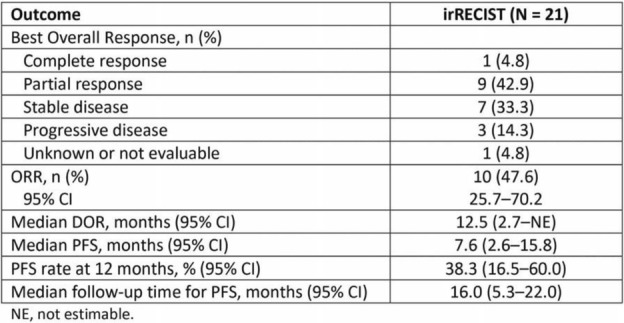



**P392**

**A phase 1b/2 trial of lenvatinib in combination with pembrolizumab in patients with non-small cell lung cancer**

Marcia Brose^2^, Nicholas Vogelzang, MD^3^, Christopher Di Simone^3^, Sharad Jain, MD^3^, Donald Richards^3^, Carlos Encarnacion^3^, Drew Rasco^4^, Robert Shumaker, PhD^5^, Corina Dutcus^5^, Daniel Stepan^5^, Matthew Guo^5^, Emmett Schmidt, MD PhD^6^, Matthew Taylor, MD^7^

^2^Abramson Cancer Center of the University, Philadelphia, PA, USA; ^3^McKesson Specialty Health, Las Vegas, NV,USA; ^4^South Texas Accelerated Research Therape, San Antonio, TX, USA; ^5^Eisai Inc., Woodcliff Lake, NJ, USA; ^6^Merck & Co., Inc., Kenilworth, NJ, USA; ^7^Oregon Health and Science University, Portland, OR, USA

**Correspondence:** Marcia Brose (Brosem@mail.med.upenn.edu) *Journal for ImmunoTherapy of Cancer* 2018, **6(Suppl 1)**:P392

**The redundant affiliation 1. Oxford PharmaGenesis, Oxford, UK as shown on the original article is no longer listed on this correction article.**

**Background**

Lenvatinib is a multikinase inhibitor of VEGFR 1−3, FGFR 1−4, PDGFRα, RET, and KIT. Pembrolizumab, an anti-PD-1 antibody, is approved as a monotherapy for previously treated patients with metastatic PD-L1–positive (tumor proportion score [TPS] ≥1%) non-small cell lung cancer (NSCLC), with an objective response rate (ORR) of 18% [1]. We report interim results of an ongoing phase 1b/2 trial evaluating lenvatinib in combination with pembrolizumab in patient with solid tumors, focusing on the metastatic NSCLC cohort.

**Methods**

In this multicenter, open-label study (NCT02501096), patients with measurable, confirmed metastatic NSCLC and ECOG performance status ≤1 received lenvatinib (20 mg/day orally) and pembrolizumab (200 mg Q3W, IV). In the phase 2 portion, patients must have had ≤2 prior lines of systemic therapy; there was no limit for phase 1b. Patients were not preselected based on PD-L1 status. Tumor assessments were performed by study investigators using immune-related RECIST (irRECIST). The phase 2 primary end point was ORR at 24 weeks (ORRWK24). Secondary end points included ORR, progressionfree survival (PFS), and duration of response (DOR).

**Results**

At the data cutoff of March 1, 2018, 21 patients were enrolled. 9 (43%) Patients were PD-L1(+) (TPS ≥1%); 5 (24%) were PD-L1(-); 7 (33%) were not tested. 3 (14%) Patients were treatment-naïve; 7 (33%), 10 (48%), and 1 (5%) patients had 1, 2, and ≥3 prior lines of systemic therapy, respectively. The primary end point of ORRWK24 was 33.3% (95% CI, 14.6–57.0). Additional efficacy outcomes are summarized in the table (Table 1). Grade 3 and 4 treatment-related adverse events (TRAEs) occurred in 10 (48%) and 1 (5%; increased aspartate aminotransferase) patients, respectively. There was 1 fatal TRAE (exsanguination; deemed “possibly related” to study treatment). The most common grade 3 TRAEs were hypertension (24%), fatigue (14%), diarrhea (14%), proteinuria (10%), and arthralgia (10%).

**Conclusions**

The combination of lenvatinib and pembrolizumab showed promising clinical activity with a manageable safety profile in previously treated patients with metastatic NSCLC who were not preselected for PD-L1 status. Further study is warranted.

**Trial Registration**

NCT02501096

**References**

Herbst RS et al. Pembrolizumab versus docetaxel for previously treated, PD-L1-positive, advanced non-small-cell lung cancer (KEYNOTE-010): a randomised controlled trial. Lancet. 2016;387(10027):1540-50.

**Ethics Approval**

This study was approved by all relevant institutional review boards.

**Table 1 (abstract P392)**. See text for description.

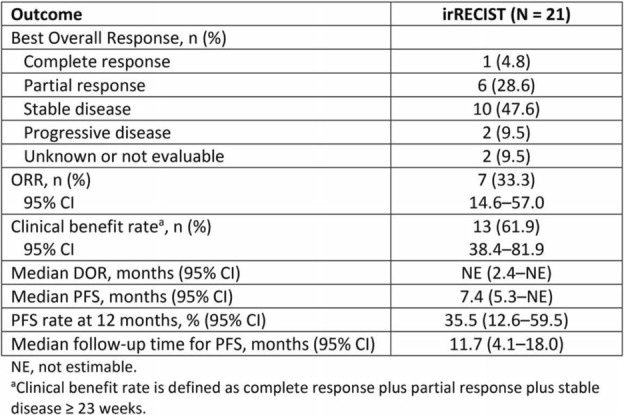



**P393**

**A phase 1b/2 trial of lenvatinib in combination with pembrolizumab in patients with urothelial cancer**

Nicholas Vogelzang, MD^2^, Carlos Encarnacion^2^, Allen Cohn^2^, Christopher Di Simone^2^, Drew Rasco^3^, Donald Richards^2^, Matthew Taylor, MD^4^, Corina Dutcus^5^, Daniel Stepan^5^, Robert Shumaker, PhD^5^, Matthew Guo^5^, Emmett Schmidt, MD PhD^6^, James Mier, MD^7^

^2^McKesson Specialty Health, Las Vegas, NV, USA; ^3^South Texas Accelerated Research Therape, San Antonio, TX, USA; ^4^Oregon Health and Science University, Portland, OR, USA; ^5^Eisai Inc., Woodcliff Lake, NJ, USA; ^6^Merck & Co., Inc., Kenilworth, NJ, USA; ^7^Beth Israel Deaconess Medical Center, Boston, MA, USA

**Correspondence:** Nicholas Vogelzang (nicholas.vogelzang@usoncology.com) *Journal for ImmunoTherapy of Cancer* 2018, **6(Suppl 1)**:P393

**The redundant affiliation 1. Oxford PharmaGenesis, Oxford, UK as shown on the original article is no longer listed on this correction article.**

**Background**

Pembrolizumab, an anti-PD-1 antibody, is approved in the secondline setting for patients (objective response rate [ORR] 21%) with advanced/metastatic urothelial cancer and in the first-line setting for patients who are ineligible for cisplatin with combined positive score ≥10 or ineligible for platinum-based chemotherapy, with ORR (overall ORR 29%) [1–3]. However, there is still an unmet need for effective therapeutic options for advanced urothelial cancer. Lenvatinib is a multikinase inhibitor of VEGFR 1-3, FGFR 1-3, PDGFRα, RET and KIT. Tyrosine kinase inhibitors, such as lenvatinib, have demonstrated activity in urothelial cancer and may reverse the immunosuppressive environment that leads to immuno-oncology (IO) therapy failure. Here we present a phase 1b/2 trial to determine the safety and efficacy of lenvatinib in combination with pembrolizumab in patients with advanced urothelial cancer.

**Methods**

In this multicenter, open-label study (NCT02501096), patients with confirmed metastatic urothelial cancer and an ECOG PS of 0 or 1 received lenvatinib 20 mg orally once daily and 200 mg pembrolizumab intravenously every 3 weeks. Patients were not preselected based on PD-L1 status. The phase 2 primary end point was ORR at week 24 (ORRwk24), as assessed by study investigators using immune-related RECIST (irRECIST). Secondary end points included ORR, duration of response (DOR), and progression-free survival (PFS).

**Results**

At the time of data cutoff (March 1, 2018), 20 patients were enrolled. 9 (45%) Patients were PD-L1(+); 5 (25%) were PD-L1(-); 6 (30%) were not tested. 4 Patients (20%) were treatment-naïve, whereas 11 (55%) and 5 (25%) patients had had 1 and 2 lines of prior anticancer therapies, respectively. No patient had received prior IO therapy. The primary end point of ORRwk24 was 25% (95% CI: 8.7–49.1). Additional efficacy outcomes are summarized in the table (Table 1). 18 (90%)

Patients experienced treatment-related adverse events (TRAEs).

Grade 3 and 4 TRAEs occurred in 5 (25%) and 5 (25%) patients, respectively. There was 1 fatal TRAE (gastrointestinal hemorrhage). The most common any-grade TRAEs were proteinuria (45%), diarrhea (40%), fatigue (30%), hypertension (30%), and hypothyroidism (30%).

**Conclusions**

The tyrosine kinase inhibitor (lenvatinib) and immunotherapy (pembrolizumab) regimen demonstrated activity in this study, which included patients receiving later-line treatment. The combination of lenvatinib and pembrolizumab deserves further investigation in patients with metastatic urothelial cancer.

**Trial Registration**

NCT02501096

**References**

1. Bellmunt J, et al. Pembrolizumab as second-line therapy for advanced urothelial carcinoma. N Engl J Med. 2017;376(11):1015-26.

2. Vuky J et al. Updated efficacy and safety of KEYNOTE-052: A single arm phase 2 study investigating first-line pembrolizumab (pembro) in cisplatin-ineligible advanced urothelial cancer (UC). J Clin Oncol. 2018;36(15 Suppl):4524.

3. Keytruda® (pembrolizumab) [package insert]. Whitehouse Station, NJ: Merck & Co., Inc.; 2017.

**Ethics Approval**

This study was approved by all relevant institutional review boards.

**Table 1 (abstract P393)**. See text for description.

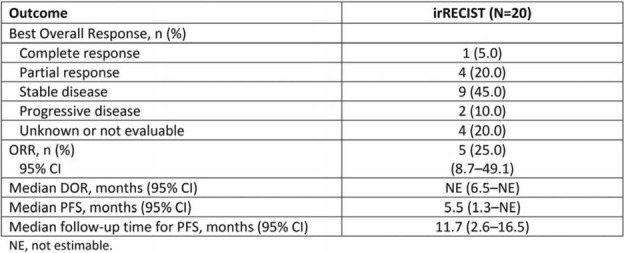



**P569**

**Novel Pharmacobiotic approach to enhance the tamoxifen efficacy using bacterial extracellular vesicles as the immunotherapy in breast cancer**

Jeongshin An, MD,PhD^1^, Yeun-yeoul Yang^1^, Won-Hee Lee^2^, Jinho Yang^2^, Jong-kyu Kim^1^, HyunGoo Kim^1^, Se Hyun Paek^1^, Jun Woo Lee^1^, Joohyun Woo^1^, Jong Bin Kim^1^, Hyungju Kwon^1^, Woosung Lim^1^, Nam Sun Paik^1^, Yoon-Keun Kim^2^, Byung-In Moon^1^

^1^Ewha Womans University, Seoul, Korea, Republic of; ^2^MD healthcare company, Seoul, Korea, Republic of

**Correspondence:** Jeongshin An (rulru81@hanmail.net) *Journal for ImmunoTherapy of Cancer* 2018, **6(Suppl 1)**:P569

**Byung-In Moon is a contributing author and has therefore been added to the author list in this correction article.**

**Background**

The anti-cancer effect of bacteria has a long history. According to Bierman et al., spontaneous remission of cancer has been observed in patients with severe bacteremia [1]. The reason was not revealed at that time, but we studied that in breast cancer. There are four main ways in which microbiota affects cancer: probiotics, prebiotics, drugs that target microbial enzymes and microbial products that have anticancer properties [2]. Among them, bacterial extracellular vesicles(EVs) are one of microbial products. In this study, we investigated the effects of bacterial EVs on the growth of breast cancer cells and tamoxifen efficacy.

**Methods**

Here, we analized microbiota of urine samples by NGS to select the target EVs that were expected to affect the growth of breast cancer cells. A total of 347 female urine samples – from 127 breast cancer patients (cancer group) and 220 normal individuals (control group) – were collected and analyzed by NGS using a universal bacterial primer of 16S rDNA. Human breast cancer cells were cultured, and the cells were treated with EVs of S. aureus and K.pneumoniae for 72 h. Real-time polymerase chain reaction (PCR) and Western blotting for signalling molecule analysis were performed after treatment of EVs in each breast cancer cell.

**Results**

There was a significant difference in the distribution of bacterial EVs between the urine samples from breast cancer patients and from normal controls. Especially, S.aureus EVs were predominant in the normal group, and K.pneumoniae was abundant in the breast cancer group. Therefore, we selected these two bacterial EVs that may have an effect on breast cancer cell growth. We found that S.aureus and K.pneumoniae EVs down-regulated cell growth in MDA-MB-231 cells. We also found that S.aureus or K.pneumoniae EVs had a synergic effect on growth inhibition of while co-treated with tamoxifen. S.aureus EVs down-regulated mRNA expression of cyclin E2 and up- regulated that of TNF-alpha which was related ERK pathway while co-treated with tamoxifen.

**Conclusions**

The anti-cancer effect of S.aureus and K.pneumoniae was initiated by its bacterial EVs and consequently inhibited the growth of breast cancer cells in triple negative breast cancer cells and improved the efficacy of tamoxifen in ER-positive cells. In the near future, we plan to conduct animal studies which are expected to further clarify the effect of bacterial EV on breast cancer. 

**Ethics Approval**

The study was approved by Ewha Womans University Medical Center‘s Ethics Board.

**Consent**

Written informed consent was obtained from the patient for publication of this abstract and any accompanying images. A copy of the written consent is available for review by the Editor of this journal.

**References**

1. Bierman, H. R. et al. Remissions in leukemia of childhood following acute infectious disease. Staphylococcus and streptococcus, varicella, and feline panleukopenias. Cancer 6, 591-605 (1953).

2. Zitvogel, L., Daillère, R., Roberti, M. P., Routy, B. & Kroemer, G. Anticancer effects of the microbiome and its products. Nature Reviews Microbiology 15, 465 (2017).

Fig. 1 (abstract P569). See text for description

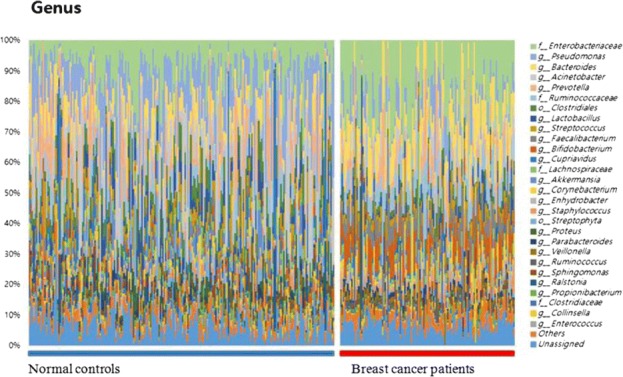



Fig. 2 (abstract P569). See text for description

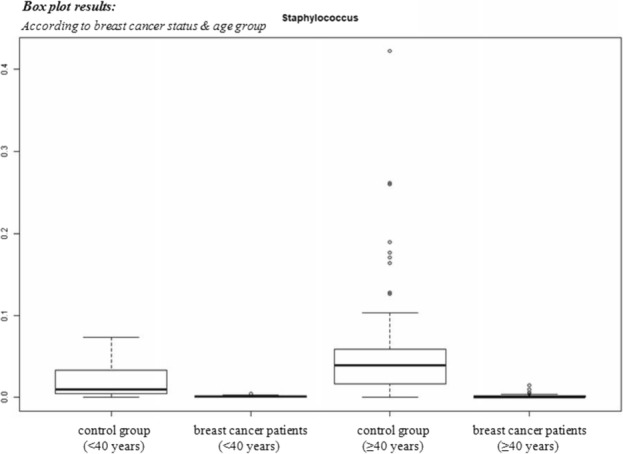



Fig. 3 (abstract P569). See text for description

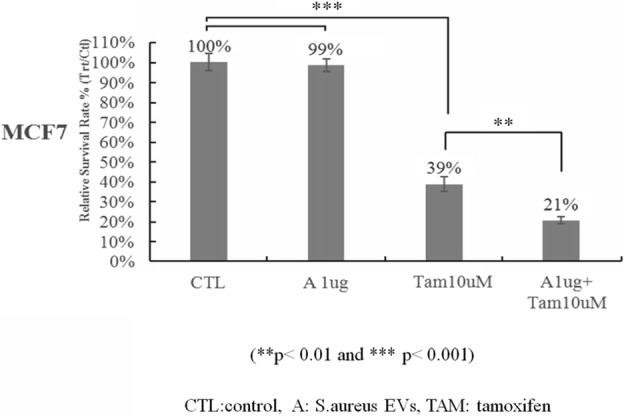



Fig. 4 (abstract P569). See text for description

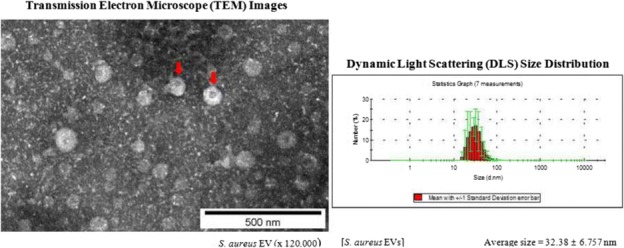



Fig. 5 (abstract P569). See text for description

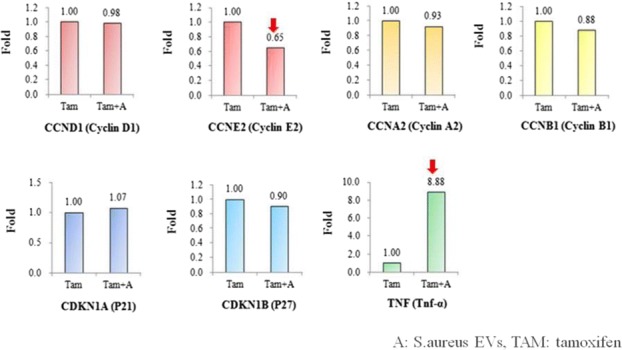



Fig. 6 (abstract P569). See text for description

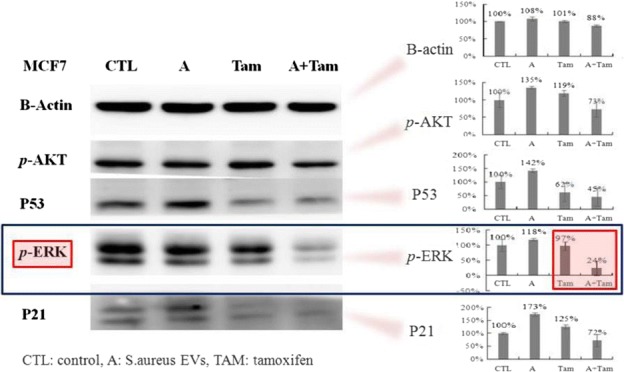



Fig. 7 (abstract P569). See text for description

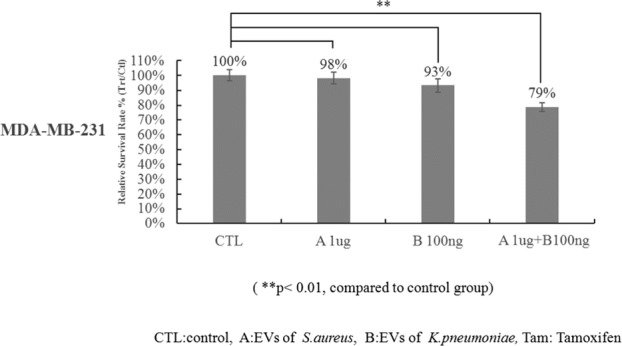



**P651**

**First-in-human study of FAZ053, an anti-PD-L1 mAb, alone and in combination with spartalizumab, an anti- PD-1 mAb, in patients with advanced malignancies**

Filip Janku, MD, PhD^2^, David Tan^3^, Juan Martin-Liberal^4^, Shunji Takahashi^5^, Ravit Geva, MD^6^, Ayca Gucalp^7^, Xueying Chen^8^, Kulandayan Subramanian^9^, Jennifer Mataraza^9^, Jennifer Wheler^9^, Philippe Bedard, MD^10^

^2^MD Anderson Cancer Center, Houston, TX, USA; ^3^National University Cancer Institute, Singapore, Singapore ^4^Vall d’Hebron Institute of Oncology, Barcelona, Spain; ^5^The Cancer Institute Hospital of JFCR, Tokyo, Japan; ^6^Tel Aviv Sourasky Medical Center, Tel-Aviv, Israel; ^7^Memorial Sloan Kettering Cancer Center, New York, NY, USA ^8^Novartis Pharmaceuticals Corporation, East Hanover, NJ, USA ^9^Novartis Institutes for BioMedical Resea, Cambridge, MA, USA; ^10^Princess Margaret Cancer Centre, Toronto, ON, Canada

**Correspondence:** Filip Janku (fjanku@mdanderson.org)

*Journal for ImmunoTherapy of Cancer* 2018, 6(Suppl 1):P651

**Janis Callister was not a contributing author and has therefore been removed from the author list in this correction article. The redundant affiliation Articulate Science as shown on the original article is no longer listed on this correction article.**

**Background**

FAZ053 and spartalizumab are humanized immunoglobulin G4 monoclonal antibodies (mAbs) that bind anti-programmed death ligand-1 (PD-L1) and programmed death-1 (PD-1), respectively. We report the dose-escalation results from an ongoing Phase I study of FAZ053 ± spartalizumab in patients with advanced malignancies, enriched for patients with chordoma, a rare subtype of sarcoma.

**Methods**

Patients received escalating doses of single-agent (SA) FAZ053 intravenously once every 3 weeks (Q3W) or 6 weeks (Q6W), or FAZ053 + spartalizumab Q3W. The primary objective was to assess the safety and tolerability of FAZ053 ± spartalizumab, and determine recommended doses for expansion (RDEs). Dose escalation was guided by an adaptive Bayesian logistic regression model following the escalation with overdose control principle.

**Results**

As of the data cutoff of March 30, 2018, 61 patients received SA FAZ053 at doses 80–1600 mg Q3W or 800–1600 mg Q6W. Most patients (n=54; 89%) received prior treatment; 1 (2%) received prior anti-PD-1. FAZ053 exposure was generally dose proportional, with terminal half-life of ~16–18 days. A dose-limiting toxicity occurred in 1 patient (Grade 4 renal failure; FAZ053 1600 mg Q6W). RDE was determined to be 1200 mg Q3W or 1600 mg Q4W. Adverse events (AEs) of all grades assessed as possibly related to treatment were reported for 33 patients (54%); most commonly (≥10%) fatigue (n=11;18%) and pruritus (n=8; 13%); 4 patients (7%) had Grade 3/4 treatment-related AEs, including elevated amylase (3%), renal failure, elevated lipase, elevated AST, and elevated blood CPK (each 2%). For these patients treated with SA FAZ053, partial responses (PRs) were demonstrated in 4 patients (7%) with chordoma, alveolar soft part sarcoma (ASPS), poorly differentiated carcinoma of scalp, and penile squamous cell carcinoma (duration of treatment 5.5–12.5 months; all with treatment ongoing). Among 5 patients with chordoma treated with SA FAZ053, 1 patient has a PR, treatment ongoing >12 months, and 4 patients have stable disease ongoing (+4% to –29%). Data for 57 patients treated with combination FAZ053 (20–1200 mg) + spartalizumab 300 mg Q3W are preliminary. Updated results and biomarker data for patients receiving SA and combination treatment, including additional patients with chordoma, will be presented.

**Conclusions**

SA FAZ053 was well tolerated and the RDE was determined to be 1200 mg Q3W. Clinical activity was observed in a range of indications including chordoma, a rare tumor without standard therapy options.

**Trial Registration**

www.clinicaltrials.gov; NCT02936102
